# RPPAML/RIMS: A metadata format and an information management system for reverse phase protein arrays

**DOI:** 10.1186/1471-2105-9-555

**Published:** 2008-12-22

**Authors:** Romesh Stanislaus, Mark Carey, Helena F Deus, Kevin Coombes, Bryan T Hennessy, Gordon B Mills, Jonas S Almeida

**Affiliations:** 1 Department of Bioinformatics and Computational Biology, The University of Texas M. D. Anderson Cancer Center, Houston, Texas, USA; 2 Department of Systems Biology, The University of Texas M. D. Anderson Cancer Center, Houston, Texas, USA; 3 Instituto de Tecnologia Química e Biológica, Universidade Nova de Lisboa, Lisbon, Portugal

## Abstract

**Background:**

Reverse Phase Protein Arrays (RPPA) are convenient assay platforms to investigate the presence of biomarkers in tissue lysates. As with other high-throughput technologies, substantial amounts of analytical data are generated. Over 1000 samples may be printed on a single nitrocellulose slide. Up to 100 different proteins may be assessed using immunoperoxidase or immunoflorescence techniques in order to determine relative amounts of protein expression in the samples of interest.

**Results:**

In this report an RPPA Information Management System (RIMS) is described and made available with open source software. In order to implement the proposed system, we propose a metadata format known as reverse phase protein array markup language (RPPAML). RPPAML would enable researchers to describe, document and disseminate RPPA data. The complexity of the data structure needed to describe the results and the graphic tools necessary to visualize them require a software deployment distributed between a client and a server application. This was achieved without sacrificing interoperability between individual deployments through the use of an open source semantic database, S3DB. This data service backbone is available to multiple client side applications that can also access other server side deployments. The RIMS platform was designed to interoperate with other data analysis and data visualization tools such as Cytoscape.

**Conclusion:**

The proposed RPPAML data format hopes to standardize RPPA data. Standardization of data would result in diverse client applications being able to operate on the same set of data. Additionally, having data in a standard format would enable data dissemination and data analysis.

## Background

Reverse Phase Protein Arrays (RPPA) provide an analytical platform with the potential to characterize proteomic pathways similarly to the use of microarrays for gene expression studies. RPPAs are a high throughput tool for probing cell or tissue lysates that quantifies levels of selected proteins for which high quality antibody exist [1]. Consequently, RPPA analysis has the potential to be a major tool in the high throughput screening of biopsies for markers of prognosis and therapy response in cancer and other complex diseases.

Protein microarrays can be classified into two groups: forward phase protein arrays (FPPA), and reverse phase protein arrays (RPPA). Forward phase protein arrays, also known as antibody arrays, employ high affinity bait molecules such as antibodies immobilized onto coated glass slides [2,3]. In reverse phase arrays, protein samples are immobilized on the slides and antibodies are used to probe the sample slides [4,5]. As a result of the differences in the immobilized medium in FPPA, one sample is probed against an array of antibodies, while in RPPA one antibody is arrayed against many samples. A major reason for its adoption could be its relative ease of producing high quality slides and its ability to quantify the amount of protein in the sample [4,6]. The ability to probe and quantify multiple samples for the expression of specific proteins in a single slide makes RPPA technology a good candidate for a high throughput analysis platform in a clinical setting.

Several analysis methods have been developed and used for quantifying signals in reverse phase protein arrays [1,5,7,8]. However, due to the large amounts and different types of data files resulting from RPPA experiments, there was a need to create an integrated platform for the management of data and integration of the available software. An integrated platform for RPPA data management becomes extremely important to organize and protect the data generated by a single experiment, and in particular helps organize both data and documentation for quality assurance purposes. Thus, at the core of the RPPA data management module is the data format known as the reverse phase protein array markup language (RPPAML). RPPAML and the data management module form the basis of an RPPA information management system (RIMS).

Consequently, three critical features are found particularly desirable in an RPPA information management system (RIMS). Firstly, the graphic visualization of the data must facilitate results reporting with specific reference to the array layout. Secondly, the data needs to be rendered in extensible markup language (XML) format (RPPAML) in order to make it easily portable to other applications and other information management systems. Thirdly, the analysis of results should include its visualization as biological networks, ideally using Cytoscape [9]. These three features outline an information system for RPPA data management that integrates processes and documents the entire experimental process. Seamless data integration and management are important success factors in proteomics experimentation and often its most time consuming [10]. RIMS hopes to provide a client side application, a repository to store the data and communication protocol based on XML for the storage and transport of the data.

## Results

RIMS is an integrated platform for reverse phase protein array data management that consists of a client side application, central repository and a communication protocol based on RPPAML data structure. The client side application consists of an upload module and a visualization module.

### Upload module

**Figure 1:**
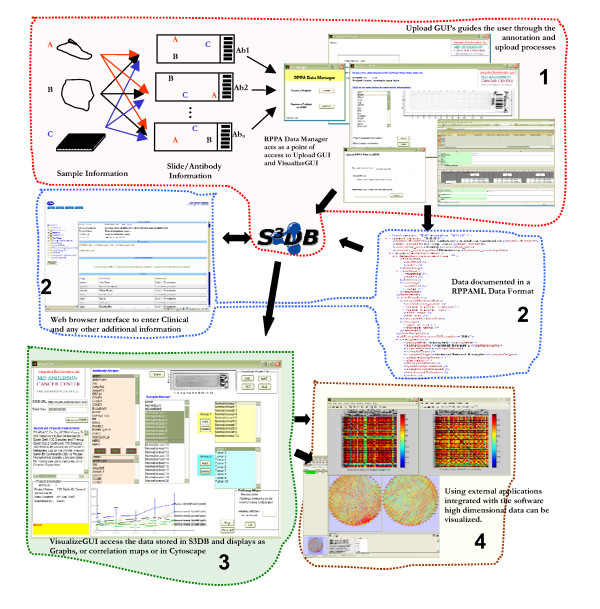
**Flow of information from experimental samples to data visualization and analysis**. The implementation architecture (shown in boxes in the figure) consists of a data management layer where data warehousing takes place (1). The next layer is the information and modeling layer where ontology and data standards can be applied to the data (2). Application integration layer provides the end user tools that interact with the data based on the ontology/standard (3). Dissemination layer (4) is used for knowledge delivery based on the data stored in the warehouse.

Data uploading and annotation is done using a separate module accessed through RPPA Data Manager GUI (please see Figure 1 for an overview of the process). Data imported is assembled with the annotation data into XML format, RPPAML (see under RPPAML metadata format for more information). The user can upload the data by pointing the application to the relevant folder and following the intuitive graphical user interface instruction. The user will be prompted to annotate the data once it has been pre-processed. Once the data is annotated, it can be saved on the local disk or uploaded to a web repository based on simple sloppy semantic database (S3DB) infrastructure [11].

RIMS is a client side application that interacts with the knowledge database (S3DB) to create a management infrastructure for RPPA data. RIMS interaction with S3DB is fully automated and location of S3DB can be distributed as long as it can be reached with a URL. Additionally, entry creation and data download is managed by RIMS software and there is no limit to the amount of data that can be uploaded or downloaded. RPPA Data Manager application manages the Upload and VisualizeGUI modules. Upload module lets the user annotate the data from what is gleaned by the software. The user enters the data through Excel templates, thereby eliminating the need to learn a new method (figure 1, box 1). Currently, the application supports MicroVigene^® ^data for conversion to RPPAML. However, as new readers for RPPA analysis become available converters for these instruments will be made available.

### Visualization module

RIMS provides many methods for data visualization, ranging from the scanned images of individual antibody arrays to the averages and standard deviations of individual samples on multiple arrays. VisualizeGUI module shows the integrated data in the context of sample names. It allows the user to export data in provided formats (RPPAML, Text, Excel, original format) for import into other applications. Additionally, the user can create correlation maps with the uploaded data. The data can also be exported to other applications for visualization (figure 1, box 3 & 4).

The RIMS client application also supports the creation of pathway maps for the selected antibody and sample lists. The generated pathways can be visualized using the popular Cytoscape tool [9] or through another application that supports the extensible graph markup and modeling language (XGMML) as an input format [12]. To generate pathway maps, the user can select corresponding groups (e.g. control (Group 1) Vs disease (Group 2)) and add them to the corresponding list boxes. Clicking the 'Go' button in the 'Pathway Maps' panel will generate the correlation maps from which pathways for the corresponding antibodies will be generated. Correlation maps and pathway maps will be displayed to the user and can then be saved or printed (Figure 1).

### RPPAML metadata format

There are three options in the current version of the client application to export the data and processed results. The original data can be exported as a Microsoft Excel document, as a text file similar to the original upload format, as a Matlab^® ^MAT file or as an XML document known as RPPAML. Exporting as an XML document is the most comprehensive option as it provides the original data with the context of its acquisition and processing, including the raw images. Since RPPAML is an application independent XML document, application developers using any programming language can access the data stored in the file. The RPPAML schema details can be found here: . A well formed RPPAML document contains minimum but sufficient information about the RPPA experiment and is defined as follows:

a) biological information: *sample biological information such as its provenance and treatment conditions, etc*.

b) antibody information: *validation information and approach*

c) detection information: *blocking, staining, amplification approach and antibody blotting information, etc*.

d) slide information: *slide preparation information such as array machine, lysate transfer method, pin or spot size, lysated amount, etc*.

e) data: *data about the experiment using the above conditions*.

Sub element <allSampleInfo> under the main element <experimentInformation> stores information about the biological sample such as its provenance, treatment conditions and other protocols (more details can be found on the website  under the schema tab). Also, under this main element sub-element <SlidePrepInfo> slide preparation information, such as array machine use and lysate transfer method used, is stored. Additionally, sub element <detection> stores information about blocking, staining and amplification approaches.

Element <arrays> describes all information about the reverse phase protein array (i.e. slide). Sub element <antibodyInfo> contains all information pertaining to an antibody used in the study. Additionally, <spotInfo> contains information about a spot in the slide and element <Img> contains the image of the slide as stored in any acceptable image file format. More information about individual elements is given on the website under the schema tab.

**Figure 2:**
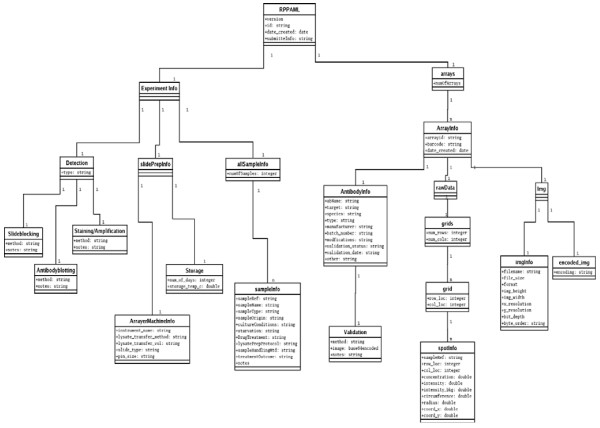
**UML representation of the RPPAML data model**. For more information on the RPPAML XML schema please go to:

The schema describing the RPPAML structure is also represented in UML notation in Figure 2. This data model is the result of interaction between experimental researchers and bioinformaticians with the purpose of capturing all the relevant information for both data management and analysis. The proposed model documents the biological context, experimental conditions and data, thereby providing the data with the provenance and context and consequently preserving the granularity of the data set. Implementation of the model was achieved through the use of XML.

### Federated repositories

RIMS uses S3DB as the data service backbone. The distributed nature of this component implies that individual users have the option of relying on locally installed S3DB deployments or using an external deployment such as the reference repository at The University of Texas M. D. Anderson Cancer Center [13]. As a consequence, individual users can access the data stored in these federated knowledge bases by simply pointing the application (RIMS) to any S3DB data service. A characteristic of S3DB semantic data services is that other data models describing complementary information can be integrated without compromising existing data [11]. This is particularly relevant for RPPA technology as new methods and improvements are devised for this young technology. However, using the proposed RPPAML data format, client applications will be aware of the context and provenance of data and provide the user with possible choices for analyzing the data.

## Discussion

The convergence of information technology and biology has resulted in an unprecedented growth in the way researchers accumulate information. The main consequence of this growth can be seen in the realm of unprocessed data. Scientific data generated by experimental biologists has changed in scale, dimensionality and diversity. Gone are the days when data could be presented in a single or multiple spreadsheets. As data increase in diversity, scale and dimensionality within and between experiments, integrating data becomes a challenge. RIMS hopes to provide a single platform for raw, analysis and eventually clinical and other relevant data, and thereby provides the researcher with an integrated view of the data and analysis results for knowledge generation.

RIMS was created as a modular management platform for RPPA data. The modularity of design and the use of XML technologies will provide the ability to add new tools. For example, tools developed in the OOMPA toolkit  package can be ported and included in RIMS. This would allow the researcher to analyze the uploaded data seamlessly. The key feature of the proposed model is the RPPAML data format that would allow researchers to describe, document and disseminate RPPA data. Additionally, the structure format described here would enable development of third party software for the analysis of RPPA data. Another feature of RIMS is the client application described in this paper, which will manage data upload and retrieval of data from S3DB data services using the RPPAML data schema. This application is also fitted with import, preprocessing and graphic visualization and export tools. These tools a) process raw data files and images into the RPPA reference data structure; b) submit the data structure to an arbitrary S3DB data service (including creating the supporting data model in that service if it does not exist); c) include basic visualization tools such as rendering the original array image and aggregating results from multiple arrays by sample identity and concentration; d) export data in a variety of formats including a comprehensive RPPAML XML format and segmentation of cross-correlation tables into a network format that is rendered using Cytoscape. Both the client and source codes of RIMS are made freely available with an open source license [14].

## Conclusion

The emergence of high throughput technologies also requires a standardized format to represent data generated from such processes. The proposed RPPAML schema provides RPPA high throughput technology with a standardized format to disseminate the data produced by such processes. Having such a format also enables different client applications to operate on the same set of data regardless of the instrument that produced the data. We hope that the proposed RPPAML format is the starting point in bringing vendors, analysts and scientists together to formulate community accepted standard for RPPA data. This in turn would enable this important technology to be widely available and useable.

## Methods

RIMS stand alone client application uses MATLAB Component Runtime (MCR, no license needed by the user). This client application uses only client side computational resources and relies on a federated S3DB data services backbone . Both components are available freely and with open source. The RIMS client is an integrated tool that guides the user from raw machine-generated data through annotation and analysis. Additionally, the RIMS tool allows the user to store the data in a federated S3DB database. The RIMS software module can be conveniently installed using Bioinformatic Station code distribution software [15]. This also allows the user to easily update/upgrade the RIMS software as new versions become available. RIMS provides tools for management, integration and analysis of RPPA data (Figure 1).

The implementation architecture (shown in colored boxes in Figure 1) consists of a data management layer where data warehousing takes place (Figure 1, box 1). The next layer is the information and modeling layer where ontology and data standards can be applied to the data (Figure 1, box 2). Information modeling is archived via the implementation of RPPAML through the use of XML. The application integration layer provides the end user with tools that interact with the data based on the ontology/standard (Figure 1, box 3). The dissemination layer (Figure 1, box 4) is used for knowledge delivery based on the data stored in the warehouse. This is done using graphs and integrating the output to external applications such as Cytoscape, etc.

## Abbreviations

RPPA: reverse phase protein arrays; FPPA: forward phase protein arrays; XML: extensible markup language; RPPAML: reverse phase protein array markup language; RIMS: RPPA information management system; XGMML: eXtensible Graph Markup and Modeling Language; MCR: MATLAB Component Runtime; S3DB: simple sloppy semantic database

## Availability and requirements

Project Name: 

Operating Systems: Windows XP

Programming Language: MATLAB

License: Open source project

Source Code: Available upon request. See website for contact details.

## Authors' contributions

RS conceived the project and served as project lead. RS and JSA provided oversight of the project. HD managed data. MC and BH acquired and processed data. RS, JSA, KC and GBM prepared the manuscript. All authors read and approved the manuscript.
